# The roles of phospholipase C-β related signals in the proliferation, metastasis and angiogenesis of malignant tumors, and the corresponding protective measures

**DOI:** 10.3389/fonc.2023.1231875

**Published:** 2023-07-28

**Authors:** Yu-Nuo Wu, Xing Su, Xue-Qin Wang, Na-Na Liu, Zhou-Wei Xu

**Affiliations:** ^1^ Department of Clinical Medical, the First Clinical Medical College of Anhui Medical University, Hefei, Anhui, China; ^2^ Department of Emergency Surgery, The First Affiliated Hospital of Anhui Medical University, Hefei, Anhui, China; ^3^ Institute of Clinical Pharmacology, Anhui Medical University, Key Laboratory of Anti-inflammatory and Immune Medicine, Ministry of Education, Anhui Collaborative Innovation Center of Anti-inflammatory and Immune Medicine, Hefei, Anhui, China

**Keywords:** PLC-β, cancer, proliferation, invasion, angiogenesis, protection

## Abstract

PLC-β is widely distributed in eukaryotic cells and is the key enzyme in phosphatidylinositol signal transduction pathway. The cellular functions regulated by its four subtypes (PLC-β1, PLC-β2, PLC-β3, PLC-β4) play an important role in maintaining homeostasis of organism. PLC-β and its related signals can promote or inhibit the occurrence and development of cancer by affecting the growth, differentiation and metastasis of cells, while targeted intervention of PLC-β1-PI3K-AKT, PLC-β2/CD133, CXCR2-NHERF1-PLC-β3, Gαq-PLC-β4-PKC-MAPK and so on can provide new strategies for the precise prevention and treatment of malignant tumors. This paper reviews the mechanism of PLC-β in various tumor cells from four aspects: proliferation and differentiation, invasion and metastasis, angiogenesis and protective measures.

## 1 Introduction

**Figure 1:**
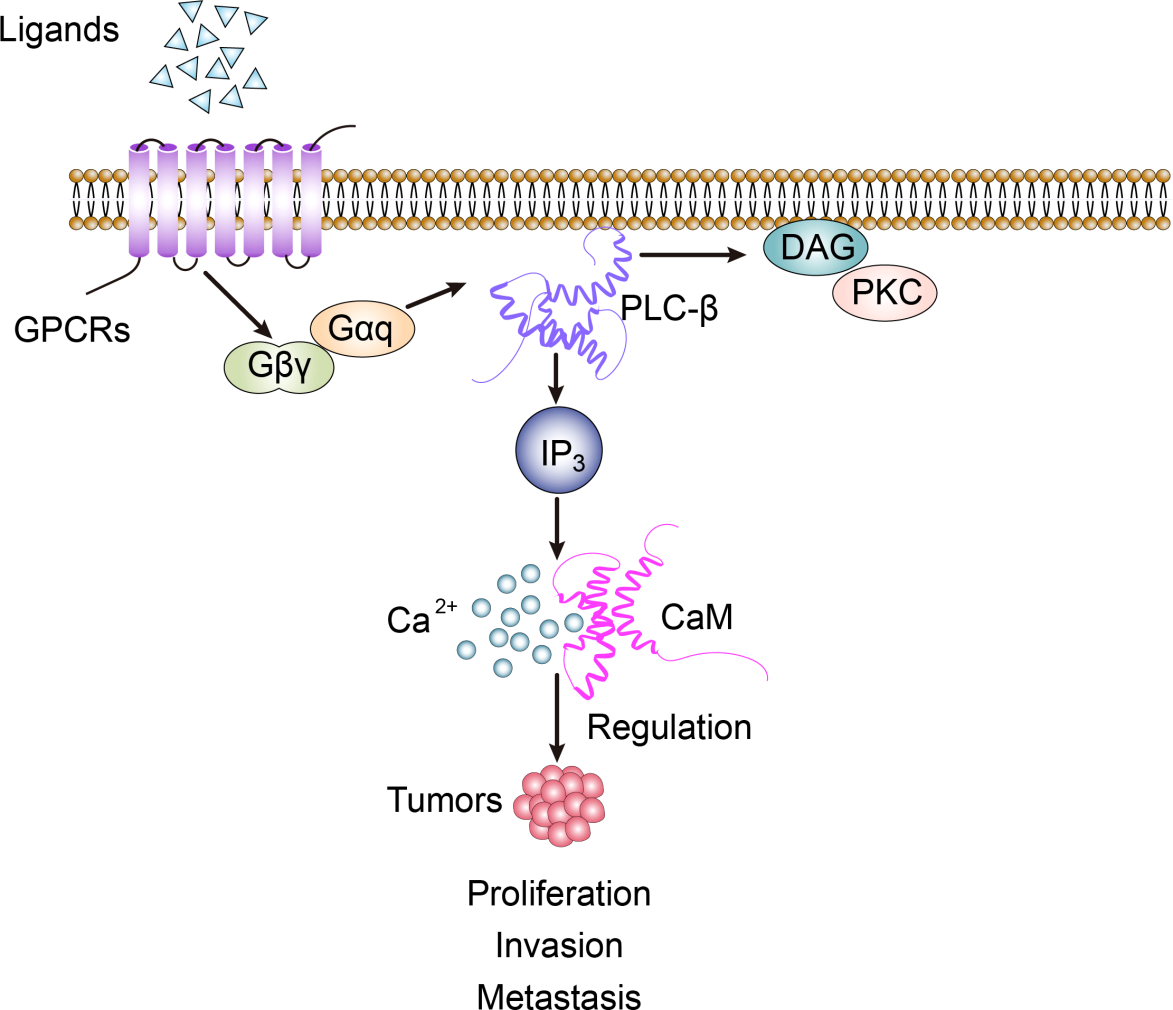
PLC-β-related signal transduction involves the regulation of biological activity of tumor cells.

PLC-β is widely distributed on the cytoplasmic membrane of eukaryotes. It belongs to β-isoenzyme of phospholipase C, which is the key enzyme in the signal transduction pathway of phosphatidylinositol. Its regulated cellular function plays an important role in maintaining homeostasis of the organism. It is an effector enzyme of G protein-coupled receptors, GPCRs, with seven transmembrane hydrophobic regions, and molecules such as bradykinin, histamine, angiotensin II, M1 muscarinic-like receptors and α1 adrenergic receptors activate PLC-β *via* Gαq of the pertussis toxin (PTX)-sensitive Gq subfamily, while M2 and M4 muscarinic-like receptors activate PLC-β *via* Gβγ of the PTX-sensitive Go/Gi subfamily (1). In the process of recognizing different subunits of G-protein, the C-terminal and N-terminal PH domain of PLC-β play an important role. The action point of PLC-β and Gαq extends at the C-terminal of the enzyme to form the tailer sequence, while the action point of PLC-β and βγ dimer is located at the N-terminal PH domain, which is the key step of exerting PLC-β’s biological effect. The basic signal pathway mediated by PLC-β is: transmembrane receptor→G-protein→PLC-β→DAG and IP3 (2). In cell signal transduction, phosphatidylinositol-4,5-bisphosphate (PIP2) can be hydrolyzed by the activated PLC-β to generate the second messenger diglyceride (DAG) and inositol-1,4,5-triphosphate (IP3). Among them, IP3 diffuses freely and binds to IP3-specific receptors, which leads to the release of intracellular Ca^2+^, while DAG activates protein kinase C (PKC) together with Ca^2+^ (**Figure 1**) (3).

PLC-β is not only distributed on the cytoplasmic membrane, but also in the nucleus and cytoplasm. PLC-β1 consist of two subtypes, 150-kDa PLC-β1a and 140-kDa PLC-β1b, which are detectable both in cytosolic and nuclear fractions (4, 5). Several studies have revealed that nuclear PLC-β1 signaling played a direct role in G1 progression by means of a specific target, i.e. cyclin D3/cdk4 (4, 6). There’s also research suggesting that nuclear PLC-β1 is a positive regulator in process of myoblast differentiation (7). Besides nuclear function, PLC-β1 has a cytosolic population that can drive the differentiation of rat pheochromocytoma cells (PC12 cells) by regulating component 3 of RISC activity (C3PO). Studies suggest that we can control the differentiation of cultured neuronal cells by down-regulating the levels of cytosolic PLC-β1 or by driving it to the membrane with stimulation of Gαq (8). Additionally, we found that reducing PLC-β1 levels increases the rate of proliferation in cells of neuronal lineage. In the early stages of differentiation, PLC-β1 binds and inhibits cyclin-dependent kinase 16 (CDK16) to promote proliferation. The bifunctional effects of PLC-β1 on differentiation and proliferation is also seen in the cultured human neuronal line SK-N-SH (9).

There are four subtypes of PLC-β, namely PLC-β1, PLC-β2, PLC-β3 and PLC-β4. They are widely distributed in human brain, breast, liver, pancreas and vascular smooth muscle tissues, and their regulated cellular functions play an important role in maintaining homeostasis (1, 2). PLC-β is involved in the process of cell proliferation, differentiation and apoptosis, and is closely related to various biological activities of tumors. In this paper, the role of PLC-β in several aspects of tumor proliferation, metastasis, angiogenesis and protective measures will be discussed separately.

## 2 Proliferation and differentiation

### 2.1 ERK/MAPK signal

Mitogen activated protein kinases (MAPKs) is a kind of serine/threonine protein kinases. Its related pathways can be activated by extracellular stimuli such as growth factors, inflammation, hormones, neurotransmitters, environment and cell stress, and then induce a wide range of intracellular reactions (10). Extracellular signal regulated kinase (ERK) was first discovered and widely studied in MAPK pathway. Mutation or abnormal activation of MAPK/ERK signaling pathway was found in more than half of cancers. ERK mainly includes five subtypes ERK1~ERK5. Among them, ERK1 and ERK2 are the two subtypes that have been deeply studied at present, and ERK1/2 plays a key role in cancer proliferation and metastasis (11, 12).

The expression of PLC-β1 in hepatocellular carcinoma (HCC) is significantly higher than that in adjacent tissues, and it is closely related to tumor stage. The results show that the high expression of PLC-β1 in cells can stimulate the phosphorylation of ERK1/2 pathway, while ERK inhibitor can inhibit the promotion effect of PLC-β1 on the growth of hepatocellular carcinoma cells. Therefore, PLC-β1 can exert carcinogenic activity by activating ERK signal in HCC cells (13). In addition, insulin-like growth factor 1 (IGF-1) induces the phosphorylation of nuclear PLC-β1 while activating nuclear ERK, and then starts the inositol phosphate cycle, which may be the loop mechanism of regulation between PLC-β1 and ERK (14, 15). miRNA-205 can also activate liver cancer stem cell subsets and maintain the characteristics of liver cancer stem cells by regulating the upstream target PLC-β1, which is closely related to the recurrence and metastasis of liver cancer (16). The expression of PLC-β1 in HCC tissues is significantly higher than that in paracancer tissues and is closely related to tumor staging. There was a positive correlation between PLC-β1 expression and advanced tumor stage, that is, PLC-β1 expression was significantly correlated with tumor T stage, and had no significant correlation with other clinical features. This suggests that high PLC-β1 expression contributes to the progression of HCC. At the same time, patients with positive tumor PLC-β1 expression had a lower OS rate than the patients with negative PLC-β1 expression. According to the above, the expression of PLC-β1 in HCC is an important prognostic factor for survival and T stage (17).

**Figure 2:**
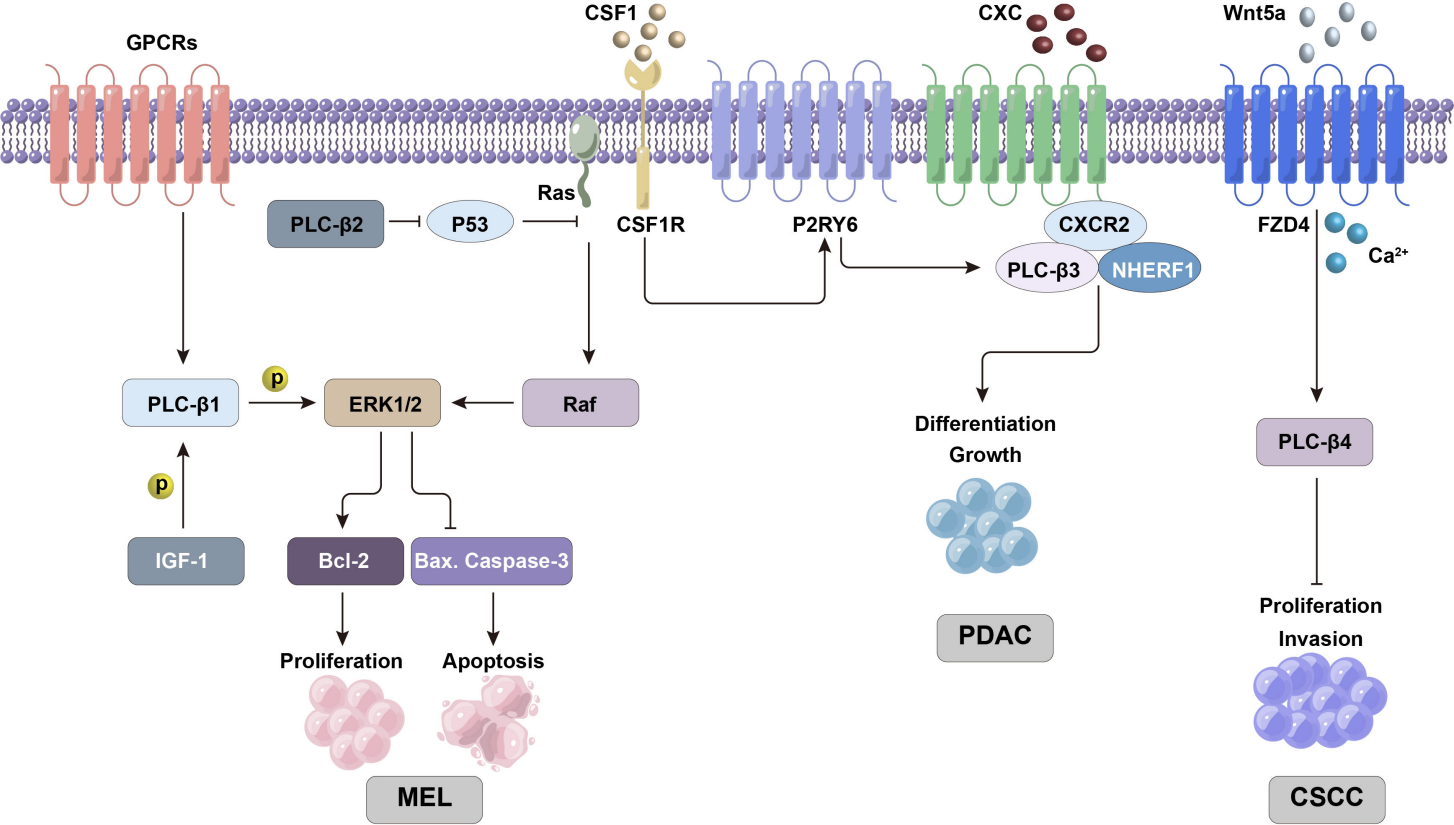
PLC-β-related ERK/MAPK and other signaling pathways are involved in regulating the proliferation and differentiation activities of tumor cells.

Melanoma is one of the highly malignant epidermal cancers. It was found that PLC-β2 was highly expressed in melanoma tissues, and its level was significantly up-regulated in human melanoma cell lines. This can affect the related biological functions of melanoma, while interfering with PLC-β2 can significantly inhibit cell viability and promote cell apoptosis (18). Ras-Raf-ERK pathway can promote cell growth, division and differentiation, and participate in cell cycle regulation, wound healing, tissue repair, cell migration and integrin signal transduction. Ras mutations are the oncogenic form in more than 15% of cancers, and the B-Raf gene (which is a member of the Raf family) is mutated in 66% of malignant melanomas. Ras can activate Raf/MEK signal in turn to up-regulate the phosphorylation level of ERK1/2, and induce the development of cancer cells (19). The mutual regulation of Ras and antioncogene p53 can affect the process of cell proliferation, apoptosis, movement, inflammatory reaction and angiogenesis, while the overexpression of PLC-β2 can significantly reduce the mRNA and protein level of p53. MAPK signal can regulate the expression of Bax, Bcl-2 and caspase-3 genes, among which Bax protein promotes apoptosis while Bcl-2 protein inhibits apoptosis, and caspase-3 is the key executor of apoptosis. It can significantly enhance the expression of caspase-3 and Bax genes and reduce the activity of Bcl-2 by interfering with PLC-β2. Therefore, down-regulation of PLC-β2 can inhibit the activation of Ras/Raf/MAPK pathway and promote the apoptosis of melanoma cells, so it is considered that PLC-β2 may be a potential target gene for controlling the proliferation of melanoma cells (**Figure 2**) (18).

### 2.2 CSF1-P2RY6 and Stat5/SHP-1 pathways

PLC-β3 is related to a specific subgroup of leukemia, namely chronic myelomonocytic leukemia (CMML). It is found that PLC-β3(-/-) mice can cause CMML-like diseases (20), which are characterized by immature and stunted granulocytes. Generally, after colony stimulating factor 1 (CSF1) binds to the receptor in hematopoietic cells, CSF1 receptor (CSF1R) triggers the activation of P2Y6 receptor (P2RY6, a GPCR in P2 receptor family), which in turn activates PLC-β3 and induces autophagy and monocyte differentiation. However, in CMML cells, the synthesis and secretion of a large number of defensin α1 (DEFA1) and defensin α3 (DEFA3) can antagonize P2RY6, thus inhibiting the monocyte differentiation induced by CSF1 and PLC-β3. Therefore, PLC-β3 knockout in hematopoietic cells can effectively repress CSF1-mediated monocyte differentiation through calcium signaling pathway (21).

In addition, the over-activation of signal transducer and activator of transcription 5 (Stat5) can also lead to CMML. Src homology region 2 domain-containing phosphatase 1 (SHP-1) is a non-receptor protein tyrosine phosphatase mainly expressed in hematopoietic cells and epithelial cells. It can dephosphorylate Stat5 to inhibit the activity of Stat5, and the existence of PLC-β3 can inhibit the malignant reaction related to Stat5 by enhancing the function of SHP-1. The same transformation mechanism has been found in other human lymphoid and myeloid malignancies (20, 22).

### 2.3 Wnt/Ca^2+^ signal

Cutaneous squamous cell carcinoma (CSCC) is a malignant tumor originating from epidermal keratinocytes or adnexal cells, which has a high incidence. Once CSCC metastasizes, it is difficult to treat and its prognosis is poor. Wnt5a (a member of Wnt protein family) can participate in the non-classical Wnt pathway through Wnt/Ca^2+^ pathway. It was found that the expression of PLC-β4 gene in this pathway decreased in cancer, but there was no significant difference in Wnt5a mRNA level. WNT5a may activate PLC-β4 by binding frizzled 4 (FZD4), and initiate the non-classical Wnt pathway to play an antioncogene-like role in CSCC. Therefore, PLC-β4 can inhibit the proliferation and invasion of CSCC cells, and its high expression can also repress the growth of tumor cells and CSCC formation in nude mice (23).

### 2.4 WNK1-PI4KIIIa and TRPC6-NFATc1 pathways

The imbalance of Ca^2+^ signal is one of the important characteristics of cancer progression. Lysine-deficient protein kinase 1 (WNK1) is the main regulator of renal ion transport, and regulates Ca^2+^ signal by stimulating phosphatidylinositol 4-kinase IIIa (PI4KIIIa) to activate Gαq coupled receptor and PLC-β signal pathway. The expression of WNK1 is directly related to the nuclear grade of clear cell carcinoma of kidney (ccRCC). Studies have shown that tumor tissues with higher Fuhrman nuclear grade express a high level of WNK1, while WNK1 protein is rarely detected in lower grade tumors. This indicates that the overexpression of WNK1 may be closely related to the pathological development of ccRCC. Functional experiments showed that WNK1 combined with PI4KIIIa activated Ca^2+^ influx mediated by transient receptor potential channel 6 (TRPC6), and Gaq coupled receptor and PLC-β signal played an important role in it (24, 25). Both the functional acquired mutation and overexpression of TRPC6 can lead to kidney diseases, such as focal and segmental glomerulosclerosis, fibrosis and renal cell carcinoma (26–29). The nuclear factor of activated T-cells cytoplasmic 1 (NFATc1) can be repressed by inhibiting the activation of TRPC6 mediated by WNK1, thus reducing the proliferation and migration activity of ccRCC cells. Therefore, TRPC6-NFATc1 pathway mediated by WNK1-PI4KIIIa through PLC-β signal may play a key role in the tumor growth of ccRCC (30), and this mechanism can provide a potential new target for the treatment of renal cell carcinoma in the future.

### 2.5 LPA

Lysophosphatidic acid (LPA) regulates PLC-β1 and PLC-β2 in different ways to enhance the proliferation and migration of intestinal epithelial cell (IEC), thus promoting wound healing and the recovery of intestinal epithelial barrier. The interaction between Gαq and PLC-β2 induced by LPA binding to LPA receptor 1 accelerated the migration of IEC cells. While LPA’s activity of promoting IEC cell proliferation is PLC-β1-dependent, involving the translocation of Gαq to the nucleus, where it interacts with PLC-β1 to change the process of cell cycle (31). In addition, LPA, through its homologous receptors (LPAR1-LPAR6), can produce a variety of cellular responses with the cooperation of PLC-β signals (32, 33), for example, LPAR2 stimulates the proliferation and migration of colon cancer cells, while the deletion of LPAR2 can inhibit the progress of colon cancer (34, 35).

### 2.6 CXC chemokine/CXCR2 and CXCL5 signals

CXC chemokine and its homologous receptor CXC chemokine receptor 2 (CXCR2) play a key role in tumor growth and angiogenesis. It has been proved that blocking the biological axis of CXC chemokine/CXCR2 can reduce the risk of malignant tumors (36–39). CXCR2 is expressed in many pancreatic ductal adenocarcinoma (PDAC) cell lines (40–43), and it is mainly involved in enhancing the proliferation and viability of cancer cells through autocrine or paracrine action (40, 43, 44). CXCR2, PLC-β3 and Na^+^/H^+^ exchanger regulatory factor-1 (NHERF1) form macromolecular complexes on the plasma membrane of pancreatic cancer cells, which functionally couples the signal cascade mediated by CXC chemokine and PLC-β3 (45). The formation of CXCR2-NHERF1-PLC-β3 complex is mediated by PDZ domain in NHERF1. Interruption of PDZ-mediated interaction can eliminate CXCR2 and PLC-β3 signals, thus inhibiting the proliferation of PANC-1 cells (human pancreatic cancer cells). This result has also been found in animal models of PDAC xenotransplantation. In addition, it was found that the destruction of CXCR2-NHERF1-PLC-β3 complex can significantly inhibit the progression of pancreatic malignant tumors *in vitro* and *in vivo* (45). Therefore, CXC chemokine/CXCR2 signaling is an important mechanism for the occurrence and development of many malignant tumors including pancreatic cancer (46–50).

Pancreatic intraepithelial neoplasia (PanIN) is considered to be the precursor of pancreatic cancer. Pathologically, PanIN lesions are classified as dysregulated ductal epithelium progressing from PanIN-1 to PanIN-3 (51, 52). Both PanIN-2 and PanIN-3 are high-grade lesions that represent the initial steps toward invasive cancer. The expression of CXCL5 in PanIN-2 and PanIN-3 was significantly higher than that of PanIN-1, indicating that CXCL5 gradually increased in the progression of precancerous PanIN lesions to invasive cancer. Patients with pancreatic cancer and high CXCL5 expression who also underwent cancerectomy had significantly lower survival rates than those without overexpression. These results indicated that CXCL5 expression was negatively correlated with tumor differentiation, clinical stage and survival rate (44).

### 2.7 Gαq-PIP2-PKC pathway

Gαq-PIP2-PKC is the core signal pathway of uveal melanoma (UM), in which PLC-β plays a key role (53). After PLC-β is activated by Gαq, phosphatidylinositol 4,5- bisphosphate (PIP2) is hydrolyzed into diacylglycerol (DAG) and inositol 1,4,5-triphosphate (IP3) (54). DAG binds to and activates a variety of proteins including protein kinase C (PKC) and Ras guanyl nucleotide releasing proteins (RasGRPs) through its C1 domain (55). IP3 can increase intracellular Ca^2+^ level and activate Ca^2+^-related signal pathways such as PKC signal. In UM microenvironment, PKC and Ras protein activator RasGRPs together activate MAPK signal, which is an important mechanism of inducing tumor cell growth. At present, it has been found that more than 90% UMs have mutations in Gαq structure, and PLC-β4 activation is the central node of carcinogenic signaling in Gαq mutated UMs (53).

## 3 Invasion and metastasis

### 3.1 Ang II/AT1R/CaM signal

Angiotensin II (Ang II), angiotensin II type 1 receptor (AT1R) and calmodulin (CaM) are important bioactive molecules that affect the formation and prognosis of HCC. Ang II can generate and up-regulate the expression of AT1R in liver cancer tissues. After AT1R is coupled with PLC-β1, it activates and promotes the change of CaM expression and conformation, thus regulating intracellular Ca^2+^ concentration and calcium-regulated kinase activity, resulting in a series of biological effects. Furthermore, PLC-β1 siRNA was used to transfect hepatocellular carcinoma cell lines, and it was found that the role of the above signal pathway was obviously weakened during the metastasis of HCC cells with low expression of PLC-β1. Similar experimental results have also been confirmed in the mouse model of hepatocellular carcinoma, which indicates that PLC-β1-related Ang II/AT1R/CaM signal is an important mechanism to promote the migration and invasion of HCC cells (3).

### 3.2 CD133, EMT markers and miR-146a

PLC-β2 is expressed in most breast tumors, and its level can affect the prognosis of patients (56). The role of PLC-β2 in different invasive breast tumors is related to molecules such as CD133 (glycosylated transmembrane protein), EMT markers and miR-146a (56–61). The expression of CD133 in breast cancer is significantly related to tumor stage, size, lymph node metastasis and sensitivity to neoadjuvant chemotherapy (60). EMT is the process of epithelial cells transforming into mesenchymal cells under certain physiological and pathological conditions, which can enhance the migration and invasion activity of cancer cells (62). MiR-146a is an antioncogene, which can inhibit the metastasis of tumor cells.

Triple-negative breast cancer (TNBC), which accounts for about 20% of all breast tumors, is a highly malignant tumor with a particularly poor prognosis. At present, there is no effective targeted therapy. In TNBC tumor-derived cells, the level of PLC-β2 is positively correlated with the motility of tumor cells (61). It was found that TNBC cells with high CD133 expression have stronger metastatic potential, while PLC-β2 level in highly invasive breast tumor-derived cells is negatively correlated with CD133 expression. That is to say, although PLC-β2 can maintain the movement of breast tumor cells, it may also down-regulate the expression of CD133 and reduce the invasiveness of cells. Therefore, PLC-β2 played a two-way regulating role in the transfer of TNBC (57, 61).

**Figure 3:**
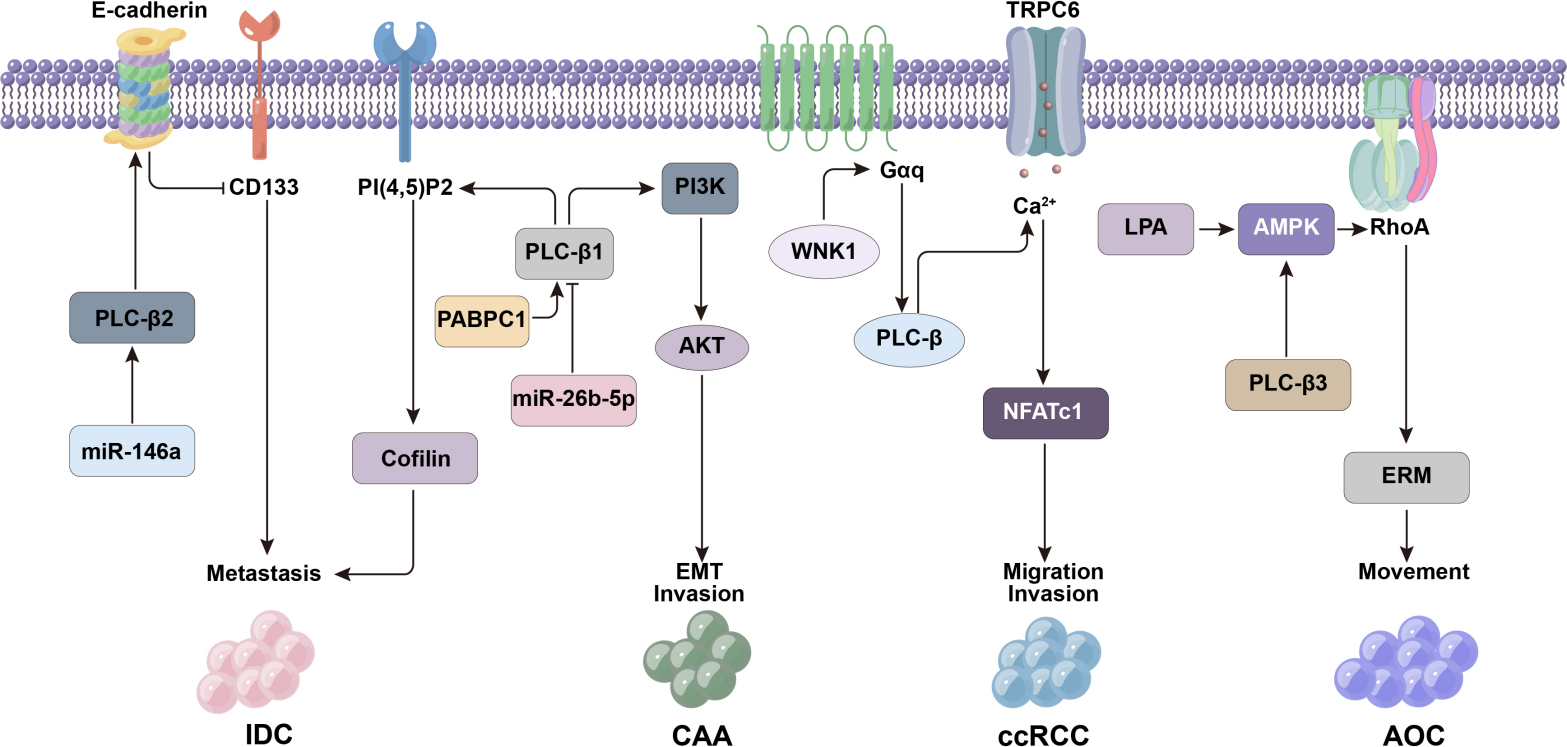
PLC-β-related signaling molecules such as CD133, E-cadherin and miR-146a affect the metastasis and invasion of tumor cells.

PLC-β2 in low-invasive breast tumor-derived cells can inhibit tumor development, but this protective mechanism is usually affected by hypoxia (57). Under hypoxia, the down-regulation of PLC-β2 is mainly related to the decrease of EMT marker E-cadherin or the up-regulation of CD133. However, the high expression of PLC-β2 in low-invasive tumor cells can also prevent the malignant progression related to hypoxia (59), that is, PLC-β2 can increase the level of E-cadherin in cells, block the up-regulation of CD133, and induce the adhesion of tumor cells to reduce their metastasis and invasion activity, thus preventing the progression of breast malignant tumors (**Figure 3**) (57).

The cancer cells of ductal carcinoma *in situ* (DCIS) are confined in the mammary duct and have not spread to the surrounding normal breast tissues. If they invade the surrounding matrix, they will develop into invasive ductal carcinoma (IDC). Up-regulation of PLC-β2 expression in DCIS can counteract the increased metastatic potential of DCIS cells caused by hypoxia. Therefore, the expression level of PLC-β2 may affect the malignant potential of DCIS, that is, the low expression of PLC-β2 makes DCIS-derived cells easy to acquire invasion characteristics. MiR-146a is a tumor suppressor miRNA in DCIS and IDC, which can reduce the migration and invasion activity of cancer cells. The expression of PLC-β2 in DCIS-derived cells is closely related to the imbalance of miR-146a. Considering that the down-regulation of miR-146a may be the basis for the ectopic appearance of PLC-β2 in tumor cells (57).

Upregulated expression levels of PLC-β2 were detected in different types of breast tumors. In addition, breast cancer cell lines with high invasive potential showed higher PLC-β2 expression levels compared to less invasive breast cancer cell lines. The expression of PLC-β2 was closely related to tumor grade, and the staining intensity increased from grade 1 to grade 3, among which grade 3 tumors showed the highest expression level of PLC-β1, and the survival rate was lower than that of other grades. This suggests that PLC-β levels are strongly correlated with poor prognosis of breast cancer (56, 63).

### 3.3 PI(4,5)P2/Cofilin

The migration of tumor cells is the key step to complete the metastasis, and phosphatidylinositol-4,5-diphosphate (PI(4,5)P2) has been proved to be an important regulator of tumor cell migration, which affects the movement ability of cells by regulating actin (64). The expression of PLC-β1 is up-regulated in highly invasive breast cancer cells, and it is related to metastasis and recurrence of breast cancer patients. It was found that PLC-β1 could hydrolyze PI(4,5)P2 to produce IP3 and DAG, which targeted to reduce the level of PI(4,5)P2 in plasma membrane, which made PI(4,5)P2 binding protein cofilin released into cytoplasm from its inactive membrane association state, thereby increasing actin in breast cancer cells to promote cell migration activity. This suggests that the PI(4,5)P2/cofilin pathway is an important mode of PLC-β1-induced cell metastasis (64).

### 3.4 PABPC1-PI3K-AKT pathway

Cholangiocarcinoma (CAA) is an epithelial malignant tumor originating from bile duct. High levels of PLC-β1 were expressed in human CCA tissues and cell lines. PLC-β1 can promote the proliferation activity of CCA cells by enhancing G1/S transition of cell cycle, and affect the movement and invasion ability of CCA cells. PLC-β1 can regulate the phosphorylation of ERK and serine/threonine kinase (AKT), and then activate phosphoinositide-3 kinase (PI3K)/AKT signal to induce CCA cells to undergo EMT. In addition, polyadenylation binding protein 1 (PABPC1) interacts with PLC-β1 can further enhance the EMT process mediated by PI3K-AKT pathway, which is an important mechanism of early metastasis of tumor cells. While PLC-β1 is the direct target of miR-26b-5p (antioncogene), which can act on PLC-β1 and prevent CCA metastasis (65).

### 3.5 WNK1-TRPC6-NFATc1 pathway

WNK1 in ccRCC cells regulates Ca^2+^ signal by activating Gαq-coupled receptor/PLC-β pathway, further mediates the activation of transient receptor potential cation channel 6 (TRPC6) and up-regulates the level of NFATc1. This is related to the metastatic potential of ccRCC cells. It was found that inhibition of TRPC6/NFATc1 signaling by cyclosporin A (CsA) or SKF96365 (calcium channel blocker) could reduce the migration activity of ccRCC cells. In addition, the invasiveness of ccRCC cells is inhibited by siRNA of WNK1 or TRPC6, while the transient overexpression of WNK1 or TRPC6 promotes cell invasion. Therefore, PLC-β related WNK1-TRPC6-NFATc1 pathway is the key mechanism to promote ccRCC transfer (30).

### 3.6 PI3K, mTOR and MAPK signals

Human colorectal tumors can express C-kit protooncogene, which has an important role in biological behaviors such as cell survival, proliferation, adhesion and chemotaxis (66). The pleiotropic function of C-kit is mainly mediated by molecular cascade of PI3K, PLC and MAPK (67–70). Existing studies have confirmed that PLC-β, mammalian target of rapamycin (mTOR) and MAPK-related signaling pathways are potential therapeutic targets for metastatic tumors (71, 72). In kidney and colon cancer cells, tumor invasion mediated by trefoil peptide family, thromboxane A2 and PAR-1 (thrombin receptor) is PLC-β or mTOR dependent (73–75). Therefore, PI3K, PLC-β, mTOR and MAPK signals affect the metastatic potential of cancer cells through their correlation with each other (76).

### 3.7 LPA/AMPK/RhoA pathway

AMP-activated protein kinase (AMPK) is an energy-sensing kinase, which can regulate the motor activity of cells. LPA induced the activation of RAS homologous gene family member A (RhoA) through AMPK, and further activated ezrin-radixin-moesin (ERM) protein to restructure the cytoskeleton, which played an important role in accelerating the migration of ovarian cancer cells. The biological function of AMPK is regulated by Ca^2+^ signal related to PLC-β3, and the ability of AMPK to promote cell migration in PLC-β3 knockout cells decreases obviously. In addition, the application of PLC inhibitor (U73122) or specific CaMKK inhibitor (STO-609) can inhibit LPA-activated phosphorylated AMPK (77). Therefore, LPA/AMPK/RhoA signaling pathway is Ca^2+^-dependent in promoting tumor metastasis.

### 3.8 PLC-β1a, PLC-β1b and ERBB4 signals

PLC-β1 is abundantly expressed in many parts of the brain, including cerebral cortex, hippocampus, amygdala, lateral septum, olfactory bulb and other regions. The signal transduction pathway mediated by PLC-β1 is related to brain development and various neurological diseases (2, 78). PLC-β1 also exists in glioma cells (4, 79), which is divided into PLC-β1a and PLC-β1b. PLC-β1a is mainly located in the cytoplasm, while PLC-β1b is mainly located in the nucleus of the cells. There is evidence that when stimulated, PLC-β1 in glioma cells will translocate into the nucleus (80). Glioma is the most common primary brain tumor, and pathological grade is the most important factor in determining the prognosis of patients. Glioma grade I was defined as the lowest invasive glioma, and grade IV was defined as the most invasive glioma type. Grades I/II and III/IV are also known as low-grade (LGG) and high-grade gliomas (HGG). Grade IV glioma, also known as glioblastoma multiforme (GBM), is the most common and malignant primary brain tumor type, accounting for 50-60% of all gliomas (81). Due to the complexity and heterogeneity of the tumor, this tumor will develop resistance to treatment and relapse quickly, and PLC-β1 is a potential prognostic factor. The expression of PLC-β1 is negatively correlated with the pathological grade of glioma, and it is a new characteristic gene in the molecular classification of high-grade glioma. Studies have confirmed that PLC-β1 gene expression is significantly reduced in all type IV gliomas (glioblastoma) compared to grade II and grade III gliomas. The data showed that the survival time of glioma patients with medium level of PLC-β1 expression was significantly longer than that of PLC-β1 down-regulated group. Compared with low-grade gliomas and healthy patients, the expression of PLC-β1 in glioblastoma samples was decreased. The experiment also confirmed that down-regulation of PLC-β1 expression in cells leads to an increase in cell migration and invasion, indicating the potential role of PLC-β1 in maintaining a normal or less invasive glioma phenotype. In addition, there is also a correlation between the expression of PLC-β1 and glioma PN signature gene ERBB4. ERBB4 protein is a tyrosine protein kinase and a member of the epidermal growth factor receptor subfamily, which will promote the pathogenesis of glioma. In the central nervous system, typical ERBB4 signaling was associated with downstream PLC and PI3K-AKT activation. In the developing brain, cleaved ERBB4 protein plays an cricial role in regulating the timing of astrogenesis. Moreover, experiments showed that PLC-β1 signal intensity was consistent with ERBB4 in low-grade and high-grade glioma tumors (78, 81).

## 4 Angiogenesis

### 4.1 VEGF and GPCR signals

Endothelial cells are the key components of neovascularization. Therefore, the proliferation and migration ability of endothelial cells is very important for angiogenesis. The proliferation and chemotaxis of endothelial cells are driven by vascular endothelial growth factor (VEGF) and basic fibroblast growth factor (bFGF). VEGF exerts its biological activity mainly by coupling VEGF receptor 1 (VEGFR1) and VEGF receptor 2 (VEGFR2). VEGF, especially VEGFR2, can induce phosphorylation of two serine residues on PLC-β3, and then enhance intracellular Ca^2+^ signal (82). It is found that cell division cycle protein 42 (CDC42) may be the downstream target molecule of VEGFR2-PLC-β3 axis to promote endothelial cell migration. In PLC-β3 knock-down cells, the expression and activity of CDC42 decreased obviously, and it could inhibit the production of endothelial cells stimulated by VEGF, resulting in abnormal development and delayed growth of tumor blood vessels (82, 83). These results suggest that PLC-β3 can be used as a therapeutic target to inhibit tumor angiogenesis.

PLC-β3 can also be activated by GPCRs, and participates in the regulation of angiogenesis of various malignant tumors. Endothelial differentiation G-protein coupled receptor 1 (EDG1) is a functional sphingosine-1-phosphate (S1P) receptor, which plays a vital role in the formation of vascular endothelial cells and the activation of PLC. It was found that EDG1 can simultaneously regulate several downstream signal pathways through Gi/o protein, including adenylate cyclase (AC) inhibition, Ca^2+^ mobilization, Ras-MAPK and PLC-β3 activation (84, 85). However, the precise molecular mechanism of PLC-β3 regulating tumor angiogenesis still needs further research and exploration (86, 87).

### 4.2 IGF2 signal

**Figure 4:**
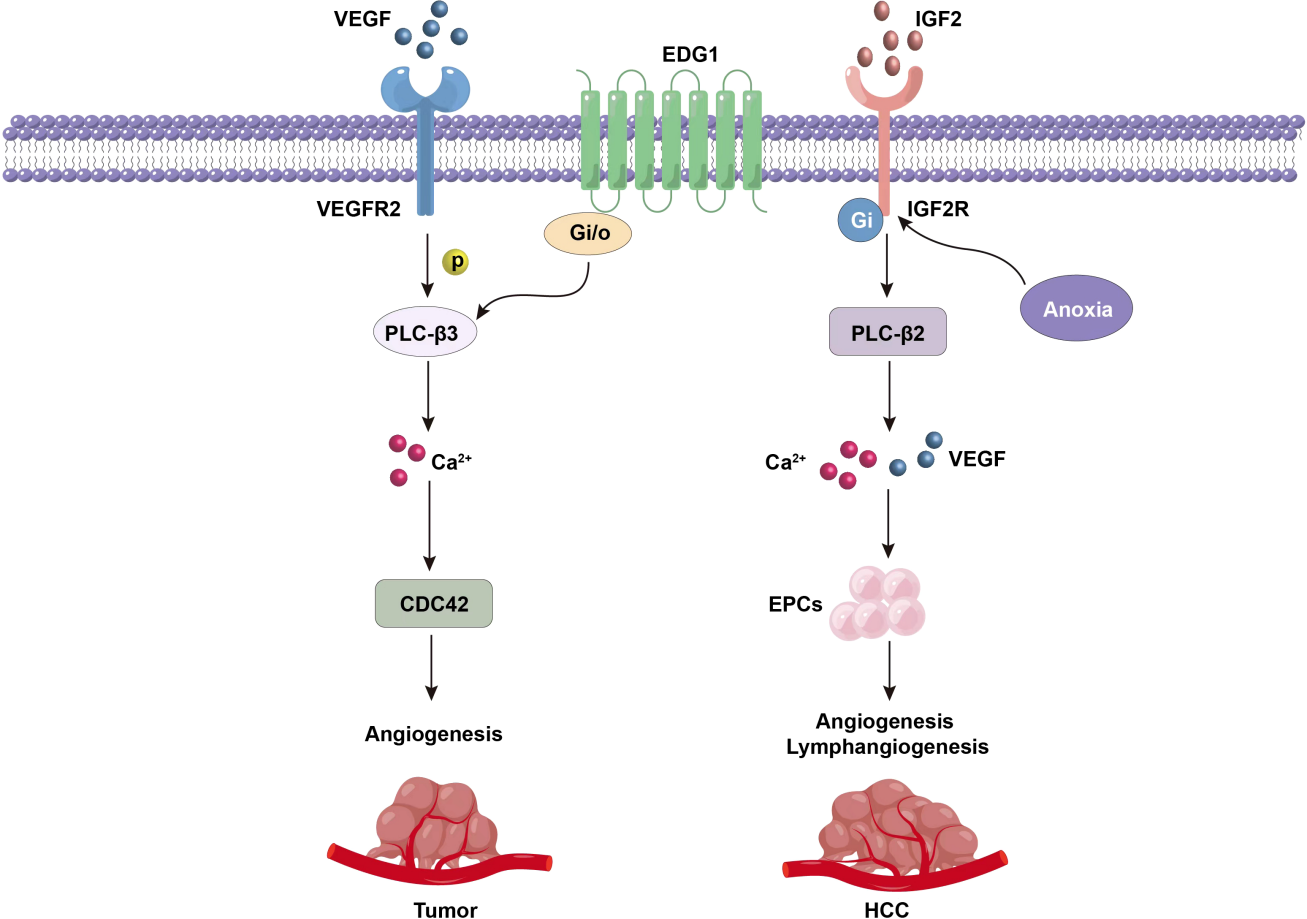
PLC-β-related VEGF and IGF2 signals regulate tumor angiogenesis.

The entry of endothelial progenitor cells (EPCs) into the neovascular region is considered to be an important step in the formation of vascular network during embryonic development. In the pathological state of tumor, the effect of EPCs on neovascularization has been generally accepted (88, 89). Insulin-like growth factor 2 (IGF2) can promote tumor angiogenesis and lymphangiogenesis by increasing EPCs recruitment (90–92). In addition, IGF2 induced by ischemia and hypoxia participates in angiogenesis of human hepatocellular carcinoma (93), and significantly increases VEGF mRNA and protein levels in tumor tissues in a time-dependent manner (90). The biological function of IGF2 is mainly realized by signal transduction of G(i) protein coupled with IGF-2 receptor (IGF2R), and intracellular Ca^2+^ mobilization induced by PLC-β2 needs to be involved (**Figure 4**) (90). Therefore, controlling the IGF-2/IGF2R signal associated with PLC-β2 can be used as a new method to treat angiogenesis-dependent tumors.

## 5 Protective measures

### 5.1 PLC-β1-related PI3K-AKT signal inhibition and PKCα signal activation

PLC-β1 can promote the proliferation and motility of CCA cells, and the CCA patients with high expression of PLC-β1 do not perform well in TNM staging, distant metastasis and survival prognosis. PLC-β1 also induced the resistance of CCA to gemcitabine combined with cisplatin, but this could be reversed by AKT inhibitor MK2206. It was found that PLC-β1-PI3K-AKT signal axis was very important for CCA development and EMT, and AKT can be used as a therapeutic target to overcome the chemotherapy resistance of CCA patients with high PLC-β1 expression, thus inhibiting the growth of tumor and obviously improving the postoperative survival of patients (65).

**Figure 5:**
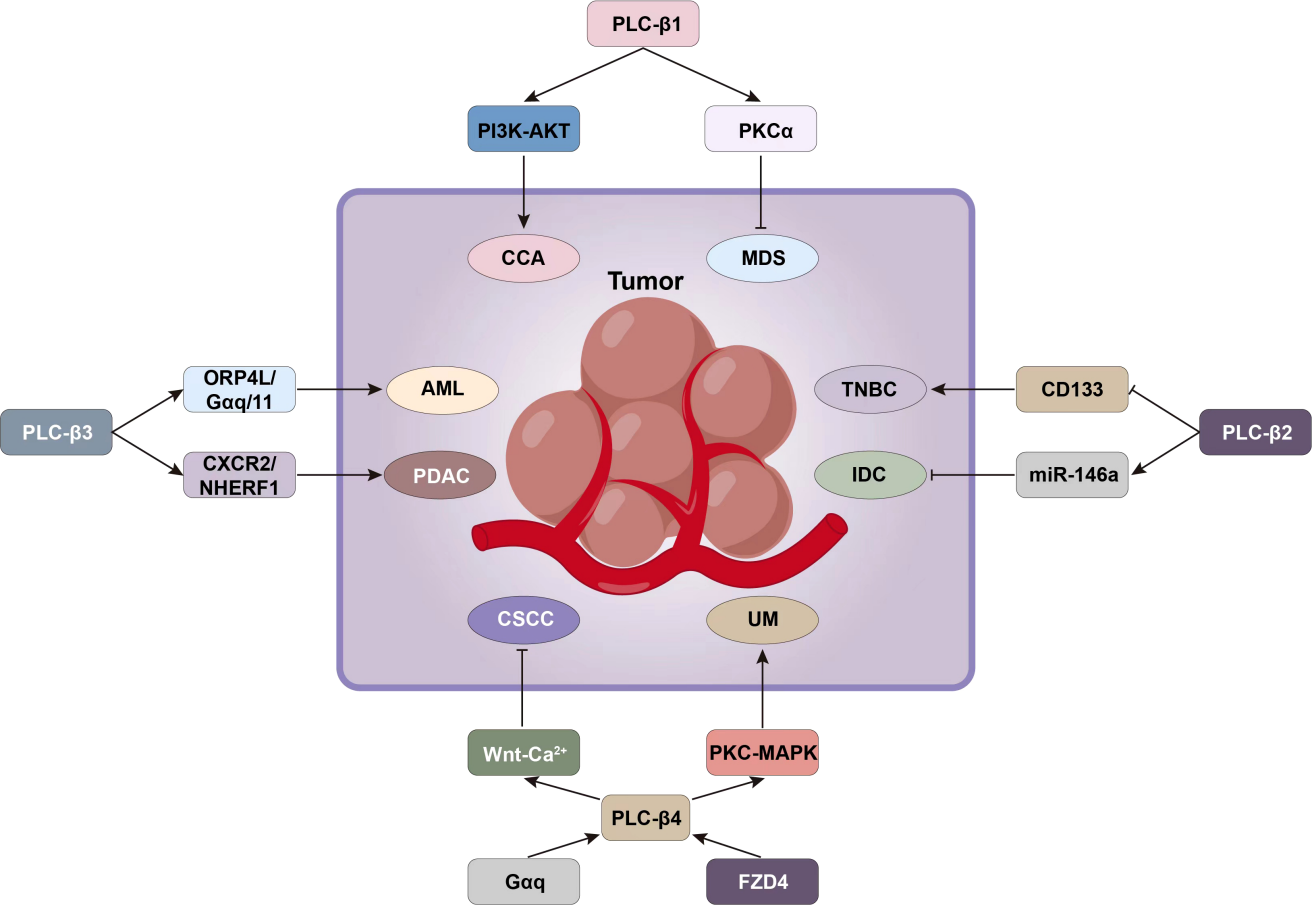
Signaling pathways such as PI3K-AKT and PKCα associated with PLC-β can be targeted for tumor intervention.

The pathogenesis of myelodysplastic syndrome (MDS) and erythropoiesis involve PLC-β1/protein kinase Cα (PKCα) signal transduction, especially nuclear PLC-β1 gene (21). PLC-β1 gene is located on the short arm of chromosome 20, and two splicing variants can be identified: PLC-β1a and PLC-β1b. Both subtypes have a nuclear localization sequence, but PLC-β1a also has a nuclear export sequence (NES). Therefore, PLC-β1a also exists in the cytoplasm, while PLC-β1b mainly exists in the nucleus (94).PLC-β1a is a negative regulator of erythroid differentiation, and which is reduced in erythropoietin-responder MDS patients and in normal hematopoietic stem cell progenitors induced to erythroid differentiation (95). Nuclear PLC-β1 specifically targets PKCα, which is related to the proliferation and differentiation of human erythroleukemia cells and primitive human erythroid cells. MDS patients with low evolutionary risk of acute myeloid leukemia (AML) and 5q chromosome deletion can be treated with lenalidomide. Lenalidomide is an immunomodulatory drug that can induce erythroid differentiation. It specifically increases the expression of PLC-β1 in cytoplasm, but it can also induce PKCα translocation to nucleus, thereby stimulating erythropoiesis (21). However, MDS patients with high risk of AML evolution usually need epigenetic therapy, which aims to inhibit the proliferation of hematopoietic stem cells. The nuclear PLC-β1 of hematopoietic cells is related to epigenetics of MDS. MDS patients can show a specific single allele deletion of the PLC-β1 gene, which can lead to a higher probability of evolution into AML, and also lead to a decrease in nuclear PLC-β1 expression levels in MDS patients. PLC-β1 is a specific molecular target of azacytidine (AZA). AZA is a demethylating drug that can accelerate tumor cell division and death by producing cytotoxicity. The cells of MDS patients showed a high level of recruitment of specific myeloid transcription factors (MZF-1) associated with the PLC-β1 promoter during azacitidine treatment. In this case, both high-risk and low-risk MDS patients who responded positively to treatment showed an early increase in the expression of nuclear PLC-β1 and PKCα, accompanied by a decrease in the specificity of PLC-β1 promoter methylation and induction of normal myeloid differentiation, leading to the improvement of clinical symptoms and the differentiation of normal bone marrow (**Figure 5**) (21).

The expression level of phosphoinositide-specific phospholipase Cβ1(PI-PLCβ1) in MDS was lower than that in normal tissue. The expression level of PI-PLCβ1 in high-risk group was lower than that in low-risk group in different types of MDS. The results indicated that the down-regulated expression of PI-PLCβ1 reflected the progression of MDS disease and could be used as a prognostic indicator of MDS (96).

### 5.2 PLC-β2/CD133 and PLC-β2/miR-146a signals

Tumor stem cell marker CD133 can predict the sensitivity of breast cancer to neoadjuvant chemotherapy drugs (61), and its level is closely related to the invasiveness of TNBC. Therefore, targeting this surface antigen may be beneficial to the treatment of TNBC (60). The regulation mechanism of PLC-β2 and CD133 in TNBC has been described above, which proves that PLC-β2 can down-regulate the expression of CD133 and reduce the metastatic potential of cells. Therefore, the intervention of PLC-β2/CD133 signal can be used as a new therapy to prevent the progression of invasive breast tumors (60). It is found that the negative correlation between PLC-β2 and miR-146a in primary DCIS cannot be detected in IDC, which indicates that the change of PLC-β2/miR-146a expression level in DCIS may constitute a molecular risk factor for IDC. Generally speaking, the down-regulation of miR-146a in DCIS without the increase of PLC-β2 will lead to the risk of malignant progression of DCIS. Evaluating the change of its level is helpful to identify whether patients have relapse tendency. Therefore, this signal pathway may become an important target for the prevention and treatment of DCIS in the future (57).

### 5.3 ORP4L/Gαq/11/PLC-β3 complex and CXCR2-NHERF1-PLC-β3 complex

AML is characterized by the rapid growth of abnormal cells accumulated in bone marrow and blood. Leukemia patients often contain leukemia stem cells (LSCs), which can produce leukemia cells and are the main cause of AML recurrence. ORP4L belongs to the oxysterol binding protein-related protein family (ORPs). It is selectively expressed in LSCs and is essential for the survival of LSCs. It was found that ORP4L transported PLC-β3 out of the nucleus of leukemia cells, and regulated PIP2 metabolism and downstream Ca^2+^ balance by forming ORP4L/Gαq/11/PLC-β3 complex, further regulating pyruvate dehydrogenase activity on mitochondria to provide oxidative phosphorylation energy for leukemia cells. This signal mechanism provided a potential intervention target for the treatment of leukemia such as AML (97).

NHERF1-mediated functional coupling of CXCR2 and PLC-β3 will form a macromolecular complex, which is essential for CXCR2 signal transduction and malignant progression of pancreatic cancer. Studies have confirmed that the decomposition of CXCR2-NHERF1-PLC-β3 macromolecules *in vitro* and *in vivo* can inhibit the growth and metastasis of pancreatic tumors. Therefore, destroying this CXCR2 complex may become an effective treatment strategy for pancreatic cancer (45).

### 5.4 Gαq-PLC-β4-PKC-MAPK and Wnt5a-PLC-β4-Ca^2+^ pathways

Gαq-PLC-β4-PKC-MAPK pathway is the core signal that affects the occurrence and development of UM (53). IP1 is a stable metabolite of the second messenger IP3, which is produced when PLC-β is activated by Gαq (98). The output intensity of PLC-β signal can be detected by the content of IP1 in cells. YM-254890 (Gαq/11 protein inhibitor) can selectively inhibit IP1 production in a dose-dependent manner, and then block PLC-β4 and downstream PKC/MAPK pathway, which inhibits the proliferation of UM cells (53). Therefore, it is possible to improve the prognosis of UM patients by selecting molecules in Gαq-PLC-β4-PKC-MAPK signaling pathway for targeted therapy.

The non-classical Wnt pathway composed of Wnt5a-PLC-β4-Ca^2+^ can reduce the proliferation and migration activity of CSCC cells, and inhibit the tumorigenicity of nude mice. Therefore, initiating the non-classical Wnt pathway is considered to play an antioncogene-like role in CSCC (23). Wnt5a can activate PLC-β4 by binding FZD4, which is the key to the activation of this signal pathway, and it also provides a new exploration direction for CSCC therapy.

## 6 Conclusion

In recent years, the regulation mechanism of PLC-β related signal pathways and its role in malignant tumors have been widely studied. They believe that PLC-β is a promising new target for the treatment of malignant tumors, and may be an important enzyme related to the diagnosis and prognosis of malignant tumors. In this paper, the regulation modes of PLC-β and its upstream and downstream signals are summarized from the aspects of proliferation and differentiation, metastasis and invasion, angiogenesis and protective measures of malignant tumors. It is found that PLC-β plays a dual regulatory role in promoting and inhibiting the development of tumors through corresponding subtypes associated with ERK/MAPK, Wnt/Ca^2+^, Gαq-PIP2-PKC, Ang II/AT1R/CaM and so on. Therefore, on the one hand, we optimistically predict that targeted intervention based on precision can open up a new clinical application direction for tumor treatment. However, on the other hand, we must realize that the safety and reliability of this treatment still depend on the accumulation of knowledge and the progress of technology.

## Author contributions

Manuscript writing: Y-NW. Literature collection: Y-NW, XS and X-QW. Figure design and painting: N-NL and Z-WX. Revision: Z-WX. All authors contributed to the article and approved the submitted version.
